# Innate immune cellular therapeutics in transplantation

**DOI:** 10.3389/frtra.2023.1067512

**Published:** 2023-03-31

**Authors:** Leah C. Ott, Alex G. Cuenca

**Affiliations:** Department of General Surgery, Boston Children’s Hospital, Boston, MA, United States

**Keywords:** cellular therapeutics, transplantation, MDSCs (myeloid-derived suppressor cells), regulatory dendritic cells, regulatory macrophages, innate lymphoid cells (ILCs), human monocyte-derived suppressor cells

## Abstract

Successful organ transplantation provides an opportunity to extend the lives of patients with end-stage organ failure. Selectively suppressing the donor-specific alloimmune response, however, remains challenging without the continuous use of non-specific immunosuppressive medications, which have multiple adverse effects including elevated risks of infection, chronic kidney injury, cardiovascular disease, and cancer. Efforts to promote allograft tolerance have focused on manipulating the adaptive immune response, but long-term allograft survival rates remain disappointing. In recent years, the innate immune system has become an attractive therapeutic target for the prevention and treatment of transplant organ rejection. Indeed, contemporary studies demonstrate that innate immune cells participate in both the initial alloimmune response and chronic allograft rejection and undergo non-permanent functional reprogramming in a phenomenon termed “trained immunity.” Several types of innate immune cells are currently under investigation as potential therapeutics in transplantation, including myeloid-derived suppressor cells, dendritic cells, regulatory macrophages, natural killer cells, and innate lymphoid cells. In this review, we discuss the features and functions of these cell types, with a focus on their role in the alloimmune response. We examine their potential application as therapeutics to prevent or treat allograft rejection, as well as challenges in their clinical translation and future directions for investigation.

## 1. Introduction

Solid organ transplantation is the only curative treatment for end-stage organ failure. While short-term patient and allograft survival have significantly improved over time, long-term allograft survival rates remain stagnant (1, 2). To prevent rejection, patients are reliant on continuous immunosuppression with medications such as calcineurin inhibitors, corticosteroids, and anti-proliferative agents. Though these regimens are critical for allograft survival, they are associated with significant morbidity including greater susceptibility to infections, chronic kidney injury, cardiovascular disease, and cancer (2, 3). New strategies to reduce or eliminate the use of these medications are needed.

Though previous efforts to promote tolerance have focused largely on the adaptive immune response, interest in innate immune cells as therapeutic targets to promote tolerance has grown, especially given recent findings that these cells can undergo non-permanent epigenetic and metabolic changes that prime their future activity in a phenomenon termed “trained immunity” (4–6). While all these cell types have been identified *in vivo*, they are not present in sufficient numbers during the alloimmune response to exert significant immunosuppressive effects, and thus must be artificially activated in the recipient, engineered *ex vivo*, or adoptively transferred following *ex vivo* expansion. In this review, we first describe these innate immune cell populations under investigation, including their endogenous features and functions, with an emphasis on their role in alloimmunity. We then discuss preclinical and clinical studies in which these cell types are modified *ex vivo* to prevent or treat allograft rejection, supporting their application as cellular therapeutics. Finally, we summarize challenges to their clinical use and future directions for investigation.

## 2. Tolerogenic innate immune cells and their immunomodulatory functions *in vivo*

### 2.1. Myeloid-derived suppressor cells

Myeloid-derived suppressor cells (MDSCs) are leukocytes that develop from immature myeloid cells in response to inflammation (7). They have been divided into two subtypes, namely polymorphonuclear MDSCs (PMN-MDCs) and monocytic MDSCs (M-MDSCs), based on their resemblance in morphology and phenotype to polymorphonuclear cells and monocytes, respectively (8). Murine PMN-MDSCs are identified as CD11b^+^ Gr-1^+^ Ly6C^low^ Ly6G^high^ cells, while M-MDSCs are defined as CD11b^+^ Gr-1^+^ Ly6C^high^ Ly6G^−^ cells (9, 10). Human M-MDSCs are designated as CD11b^+^ CD14^+^ CD15^−^ HLA-DR^low/−^ cells, while PMN-MDSCs are defined as CD11b^+^ CD15^+^ CD14^−^ cells (8, 9). The ratio of these two subtypes varies by inflammatory condition and while the optimal proportions of each to effectively suppress the alloimmune response remains unclear, evidence suggests a more critical role for M-MDSCs in tolerance induction (11–14). MDSCs were first described in cancer biology, in which they play a deleterious role suppressing anti-tumor T cell responses and creating an immunosuppressive milieu for unopposed tumor growth (15–18). Additional research has illustrated their involvement in other inflammatory conditions, including autoimmunity, trauma, sepsis, and allograft rejection (7, 11, 19–23). MDSCs interact with their primary targets, natural killer (NK) cells and effector T cells, through cell-cell interactions and signaling with soluble factors (22, 24, 25). Firstly, MDSCs express Programmed Cell Death Ligand 1 (PD-L1), activating regulatory T cells (Tregs) and suppressing activated T cells by binding their cognate Programmed Cell Death Protein 1 (PD-1), which has been shown to be necessary for their immunosuppressive effect in a murine model of islet transplantation (14, 26–28). Colony stimulating factor 1 receptor (CSF1R) is also critical to MDSC functions, binding colony stimulating factor (CSF) to regulate their expansion and migration (29, 30). CSF/CSF1R signaling has been shown to recruit MDSCs to the tumor microenvironment and promote upregulation of PD-L1, reducing the efficacy of chemotherapy, radiation, and checkpoint immunotherapy in various cancers (31–33).

**Figure 1:**
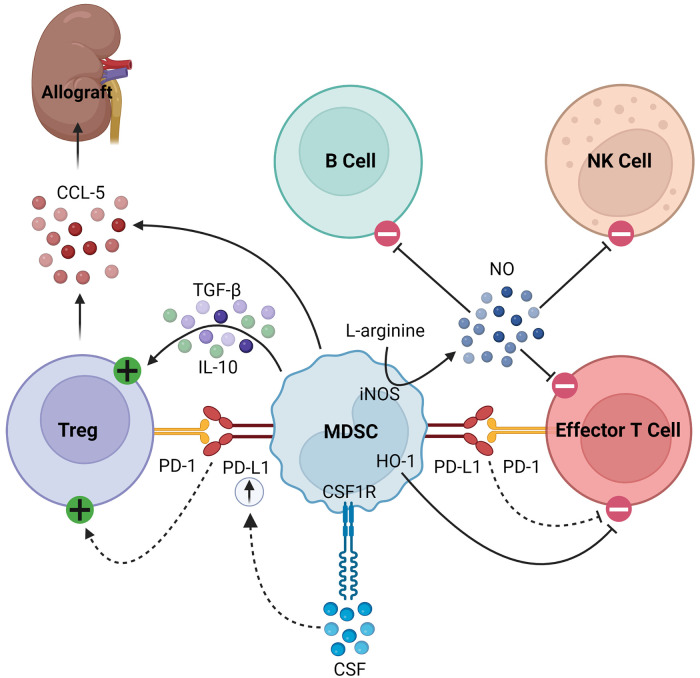
Immunosuppressive mechanisms of MDSCs. MDSCs express PD-L1, which activates Tregs and inhibits effector T cells by binding its cognate PD-1. In response to CSF/CSF1R signaling, MDSCs upregulate PD-L1. They express HO-1, which similarly inhibits effector T cells. MDSCs express iNOS which consumes L-arginine to produce NO, the latter of which then inhibits B cells, NK cells, and effector T cells. Finally, they produce TGF-β and IL-10 to activate Tregs and CCL-5 to recruit Tregs to the allograft.

MDSCs also secrete soluble factors that modulate immune responses. For example, they produce nitric oxide (NO) via inducible nitric oxide synthase (iNOS), which inhibits the expansion, differentiation, and effector functions of NK cells, B cells, and T cells (14, 21, 22, 34–39). The synthesis of NO simultaneously depletes L-arginine, a critical substrate for T cell expansion (38, 39). MDSCs also consume L-arginine through the expression of arginase-1 (Arg-1), producing urea and ornithine (34). Furthermore, MDSCs express heme oxigenase-1 (HO-1), which suppresses T cells and mediates delays in skin graft rejection (40). MDSCs produce transforming growth factor-β (TGF-β) and interleukin (IL)-10 in response to interferon-γ (IFN-*γ*) signaling, which results in downstream activation of Tregs (18, 36). Finally, in models of kidney and heart transplantation in rats, MDSCs were found to play a unique role in trafficking Tregs to the allograft from secondary lymphoid organs by creating a C-C chemokine ligand 5 (CCL5) gradient between the graft and periphery, as summarized in Figure 1 (41).

Correlational studies have demonstrated that MDSCs develop in patients following solid organ transplantation and support a potential role for these cells in promoting tolerance (11, 42–44). MDSCs were shown to expand in the peripheral blood of patients following renal transplantation and correlated with their level of circulating Tregs, but more contemporary studies did not identify them in the peripheral blood or within the allograft of such patients using single-cell cytometry by time-of-flight and single cell RNA sequencing, respectively (11, 44–46). In acute T cell-mediated rejection, greater numbers of circulating MDSCs were associated with superior allograft survival and function (42). Finally, in lung transplant patients with stable allograft function, circulating levels of MDSCs were higher than in those with chronic allograft dysfunction (43). Overall, correlational studies in transplant patients suggest a promising role for MDSCs in preventing or treating allograft rejection, further supported by preclinical studies that will be discussed in the following section.

### 2.2. Regulatory dendritic cells

Dendritic cells (DCs) are a heterogeneous population of myeloid antigen presenting cells (APCs) that regulate immunity and maintain self-tolerance under homeostatic conditions (47, 48). Human DCs are divided into conventional DCs (defined as CD11c^+^ HLA-DR^+^ cells) and plasmocytoid DCs (defined as CD11c^−^ HLA-DR^+^ CD123^+^ cells) (49). Regulatory DCs (regDCs or tolerogenic DCs) were first described in 1996 and so named for their tolerogenic properties *in vivo*, but it remains uncertain whether they represent an alternatively activated population or their own unique lineage (50, 51). Human regDCs are defined as CD11c^low^ CD11b^high^ CD14^+^ HLA-DR^+^ MHCII^low^ CD86^low^ DCs with low expression of co-stimulatory molecules (including CD40, CD80, and OX40L), MHCI, and adhesion proteins, but high expression of co-inhibition ligands (such as PD-L1) and death-inducing proteins [such as Fas ligand (FasL)] (52–55).

**Figure 2:**
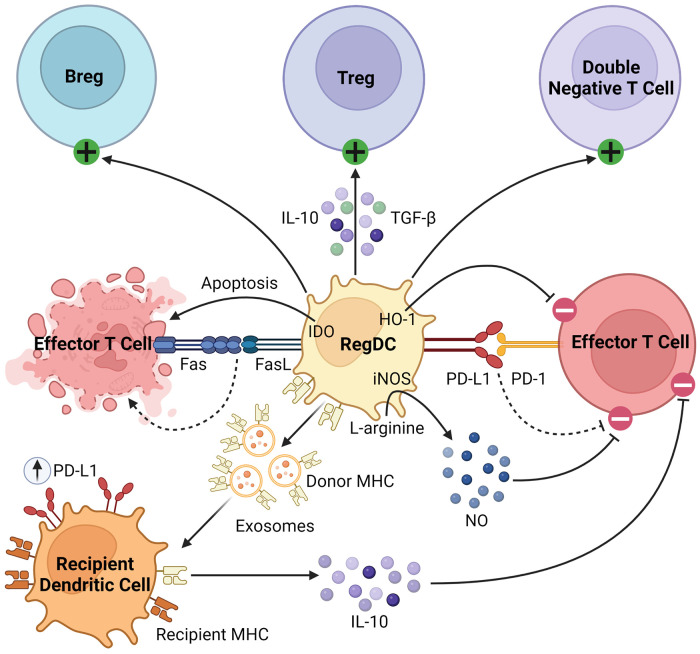
Immunosuppressive mechanisms of regDCs. RegDCs express PD-L1 and FasL to inhibit or delete effector and memory T cells through direct cell-cell contact. They also inhibit effector T cell activation through HO-1 and IDO signaling. They activate and promote the differentiation of Bregs, Tregs, and double negative T cells, and secrete anti-inflammatory cytokines including TGFβ, NO, and IL-10. Finally, donor regDCs release exosomes carrying donor MHC, creating “cross-dressed” recipient DCs via trogocytosis. These recipient DCs then upregulate inhibitory cell surface receptors (such as PD-L1) and secrete anti-inflammatory cytokines (such as IL-10) to further inhibit alloreactive T cells.

Beyond their well-described role as APCs, regDCs induce tolerance through several mechanisms, as summarized in Figure 2. They inhibit effector T cells through direct cell-cell interactions, triggering clonal deletion and anergy (56, 57). They upregulate various pro-apoptotic or immunomodulatory signals, including FasL, PD-L1, and indoleamine 2,3-dioxygenase (IDO), to delete or inhibit the proliferation of naïve and memory T cells (58–60). Like MDSCs, regDCs upregulate HO-1, which suppresses proinflammatory cytokine production and alloreactive T cell expansion (61–63). They drive the differentiation and activation of immunosuppressive immune cells, namely regulatory B cells (Bregs), Tregs, and double negative T cells (64–67). They secrete various anti-inflammatory soluble factors and cytokines such as NO, TGFβ, and IL-10 (60, 68, 69). Finally, dendritic cells release exosomes, or membrane nanovesicles carrying MHC molecules, which were recently shown to induce and sustain peripheral tolerance in various animal models of transplantation (70–73). These donor-derived exosomes are believed to promote tolerance through trogocytosis, creating “cross-dressed” recipient DCs that upregulate inhibitory cell surface markers (such as PD-L1) and cytokines (such as IL-10), suppressing the alloimmune response and prolonging allograft survival (74, 75). Based on these immunoregulatory properties of regDCs, as well as the observation that deletion of DCs induces spontaneous autoimmunity, they have been investigated in the setting of transplantation in both preclinical models and clinical trials, which will be discussed in detail in the next section (48).

### 2.3. Regulatory macrophages

Macrophages are a heterogenous, highly plastic population of myeloid cells that play diverse roles in health and disease states, including phagocytosis, antigen presentation, tissue repair, and angiogenesis (76, 77). They can be classified into one of three subtypes: classically activated (M1) macrophages, alternatively activated (M2) macrophages, and regulatory macrophages (Mregs) (78). While M1 macrophages exhibit marked proinflammatory and bactericidal properties and M2 macrophages participate in wound healing and angiogenesis, Mregs have garnered particular attention in the field of transplantation for their robust inhibition of T cells (55, 77, 79–82). Human Mregs are defined as CD14^−/low^ CD16^−^ HLA-DR^+^ CD40^−/low^ CD80^−/low^ CD83^−^ CD163^−/low^ TLR2^−^ TLR4^−^ cells (83–86). Murine Mregs are identified as CD11b^+^ CD11c^+^ CD14^+^ MHCII^int^ CD40^−^ CD80^int^ CD86^−^ CD169^+^ CD204^+^ CD206^−^ TLR2^−^ TLR4^−^ cells (55).

**Figure 3:**
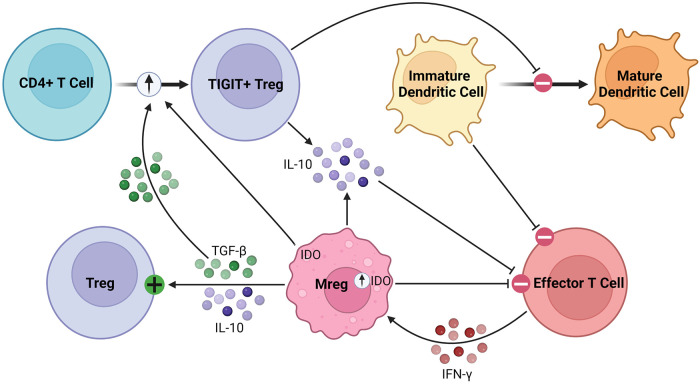
Immunosuppressive mechanisms of Mregs. Mregs express IDO in response to IFN-γ signaling, which then inhibits effector T cell proliferation. They secrete TGF-β and IL-10 to activate and promote the expansion of Tregs. Additionally, Mregs promote the conversion of allogenic CD4^+^ T cells to inhibitory TIGIT^+^ Tregs through the TGF-β and IDO signaling pathways, among others. The TIGIT^+^ Tregs secrete IL-10 and arrest dendritic cell maturation, the latter of which promotes allogenic T cell anergy or deletion through the indirect allorecognition pathway.

As robust APCs with high expression of costimulatory molecules and anti-inflammatory cytokines, Mregs exert their immunosuppressive effects in various inflammatory conditions (87, 88). Parasites have been shown to promote Mreg induction, leading to impaired anti-parasitic immunity and chronic infection (89, 90). In various tumor models, conventional DCs are converted to Mregs which subsequently suppress inflammation (91). Mregs attenuate inflammation through suppression of activated T cells, stimulation of Tregs, and production of anti-inflammatory soluble factors, such as IL-10 and TGF-β, as illustrated in Figure 3 (92). Following IFN-γ signaling, Mregs produce IDO in humans and NO in mice to block T cell proliferation (84, 86). Secondly, Mregs suppress inflammation by promoting Treg expansion through TGF-β signaling and converting allogenic CD4^+^ T cells to inhibitory TIGIT^+^ FoxP3^+^ Tregs, the latter enhancing IL-10 production (93–95). Furthermore, TIGIT^+^ Tregs arrest DC maturation, resulting in allogenic T cell anergy or deletion via the indirect allorecognition pathway (55, 93). These findings have been correlated *in vivo*, with humanized mice demonstrating elevated levels of circulating TIGIT^+^ Tregs following Mreg administration (93, 94). Overall, this suggests a feed-forward mechanism between Mregs and Tregs to maintain an immunosuppressive environment lasting beyond the lifespan of adoptively transferred Mregs, which is promising for their application in solid organ transplantation (96). They seem to exert lasting effects on allogenic T cells, as any remaining after co-culture have significantly attenuated IFN-γ and IL-2 production on repeat stimulation (83).

Beyond their immunosuppressive effects, Mregs limit fibrosis by suppressing M2 macrophage arginase production and fibroblast proliferation (97). Finally, they stimulate angiogenesis following hypoxia through upregulation of angiogenic proteins, suggesting they may limit allograft fibrosis and ischemia-reperfusion injury (IRI) (97, 98). The robust capacity of Mregs to suppress inflammation and promote tissue repair highlights why they, along with regDCs, have been the most extensively studied and developed as innate immune cellular therapeutics to date. Data regarding their application in preclinical and clinical studies will be discussed in detail in the next section.

### 2.4. Natural killer cells

NK cells are cytotoxic lymphoid cells known for their roles in anti-tumor and anti-viral immunity (99). Their cytotoxic activity does not require prior sensitization against a target and can be activated by cells lacking self MHC, while their inhibitory receptors recognize self MHC to prevent autologous cell death (100). Murine NK cells are identified as CD3^−^ TCR^−^ cells that express either DX5 or NK1.1 (101, 102). Human NK cells are defined as CD3^−^ CD56^+^ NKp46^+^ cells, then can be further divided into subtypes of high density CD56^bright^ cells and low density CD56^dim^ cells (103). CD56^dim^ cells express CD16, exhibit cytotoxic activity, and are found in the circulation, while CD56^bright^ cells lack CD16 expression, localize to secondary lymphoid organs and peripheral tissues, and modulate the immune response through the production of cytokines, including IFN-γ and TNF-α (104, 105).

NK cells were initially shown to play immunoregulatory roles in other disease states such as bacterial infections and tumor immunization, in which they delete immature dendritic cells to prevent excessive inflammation (106, 107). Over the years, various immunoregulatory mechanisms of NK cells have been elucidated, including cytotoxic killing of APCs and effector T cells, activation of Tregs, cell surface receptor signaling, and cytokine production (108, 109). NK cells can inhibit the alloimmune response by killing donor DCs using perforin and granzyme, suppressing downstream activation of host alloimmune CD8^+^ T cells (110, 111). They have also been shown to delete recipient DCs to prevent presentation of allograft antigens (112). Alternatively, they can target activated allogenic CD4^+^ and CD8^+^ effector T cells for cytotoxic killing (113, 114). Beyond their cytotoxic activity, NK cells may shape the immune response via cytokine production in response to damaged or infected cells (115, 116). NK cells can produce IL-10, which promotes Th2 polarization of inflammation and Treg expansion (117). They may also activate Tregs in response to TGF-β signaling in the inflammatory milieu (118, 119). Furthermore, Deniz et al. found that a subset of NK cells in peripheral blood monocytes (PBMCs) isolated from healthy human subjects could suppress CD4^+^ T cell responses in an antigen-specific manner via IL-10 secretion (120).

Their immunosuppressive capacity can also be mediated by cell surface receptor signaling (121–124). Transplant tolerance induced by costimulatory blockade requires the presence of NK cells and their expression of the NKG2D receptor, which attenuates IFN-γ secretion and degranulation (121, 122). NK1.1^+^ cells in the liver were found to upregulate NKG2D expression to trigger IL-22 secretion, which then promoted allograft tolerance by limiting inflammation (123). Similar to NKG2D, lower expression of CD16 on CD56^dim^ NK cells was associated with decreased expression of IFN-γ and perforin (124). Finally, NK cells express killer-like immunoglobulin receptors (KIRs) that bind MHCI molecules, and a subset of KIRs trigger downstream inhibitory signaling that suppresses NK cell activity (125). These cell surface receptors are notably expressed by uterine NK cells during pregnancy, which are known to regulate inflammation at the maternal-fetal interface and induce a tolerogenic environment (126, 127). In the presence of fetal HLA-C2^+^ cells, KIR2DS1^+^ uterine NK cells have been shown to promote the expansion of inhibitory monocytes expressing IDO, activate Tregs, and target effector T cells for apoptosis (118, 128–130). Furthermore, HLA-E expression on fetal cells has been shown to suppress NK cells via signaling through NKG2A (126).

While there are no clinical trials of NK cells in transplantation to date, correlational data suggests they play a role in allograft tolerance (124, 131–134). Compared to those with rejection, tolerized liver transplant patients have elevated numbers of circulating NK cells (131). In a study of kidney transplant patients with stable allograft function, CD56^dim^ NK cells downregulated NKp46 and perforin compared to healthy controls (124). Patients with operational tolerance no longer requiring immunosuppression also demonstrated lower CD16 expression on these cells, which was associated with reduced IFN-γ secretion and cytotoxic activity (124). Downregulation of CD16 appears to promote tolerance, while upregulation has been associated with antibody-mediated rejection (124, 132). NK expression of KIR2DL1 and KIR3DL1, two subtypes of inhibitory KIRs, was ubiquitous in tolerized deceased donor kidney transplant patients, and the absence of both plus their cognate HLA ligands was associated with a heightened risk of chronic rejection, suggesting they may play an important role in tolerance induction (124, 133). Finally, a subset of these cells termed regulatory NK cells are though to suppress the alloimmune response through similar mechanisms as the uterine NK cells described above (135, 136). These regulatory NK cells express CD16 and/or CD56 and secrete perforin, granzyme, IFN-γ, and IL-10 (134). Indeed, such cells have been detected in the blood of renal transplant patients after achieving stable, durable allograft function (134). Based on the immunosuppressive properties of NK cells and available correlational data in transplant populations, the capacity of NK cells to inhibit alloimmunity has been investigated in preclinical models, which we summarize in the next section.

### 2.5. Innate lymphoid cells

Innate lymphoid cells (ILCs) are a family of lymphoid cells that play important roles in both homeostasis and disease (137–142). They are found in various tissues throughout the body but are particularly enriched at mucosal barriers (143–150). Following their activation by lipid mediators, alarmins, or neuropeptides, ILCs regulate inflammation and adaptive immunity at these barriers (142, 151). The ILC family encompasses NK cells and three subtypes of ILCs: group 1 (ILC1), group 2 (ILC2), and group 3 (ILC3). ILC1s, ILC2s, and ILC3s can be distinguished based on their transcription factor, cytokine, and cell surface marker expression, and are functionally homologous to the Th subsets of the adaptive immune system (152–154). ILC2s are defined as lineage^−^ CD127^+^ c-kit^+^ Sca1^+^ ST2^+^ GATA3^+^ cells (154). They have been extensively studied in allergic airway inflammation and helminth infections, in which they promote a type 2 inflammatory response (143–146, 148, 155). They have garnered particular attention, however, as a potential therapeutic target for preventing or treating allograft rejection based on their newly characterized roles in tissue repair, suppression of damaging type 1 inflammation, and induction of other immunosuppressive cells (146, 154, 156–164).

**Figure 4:**
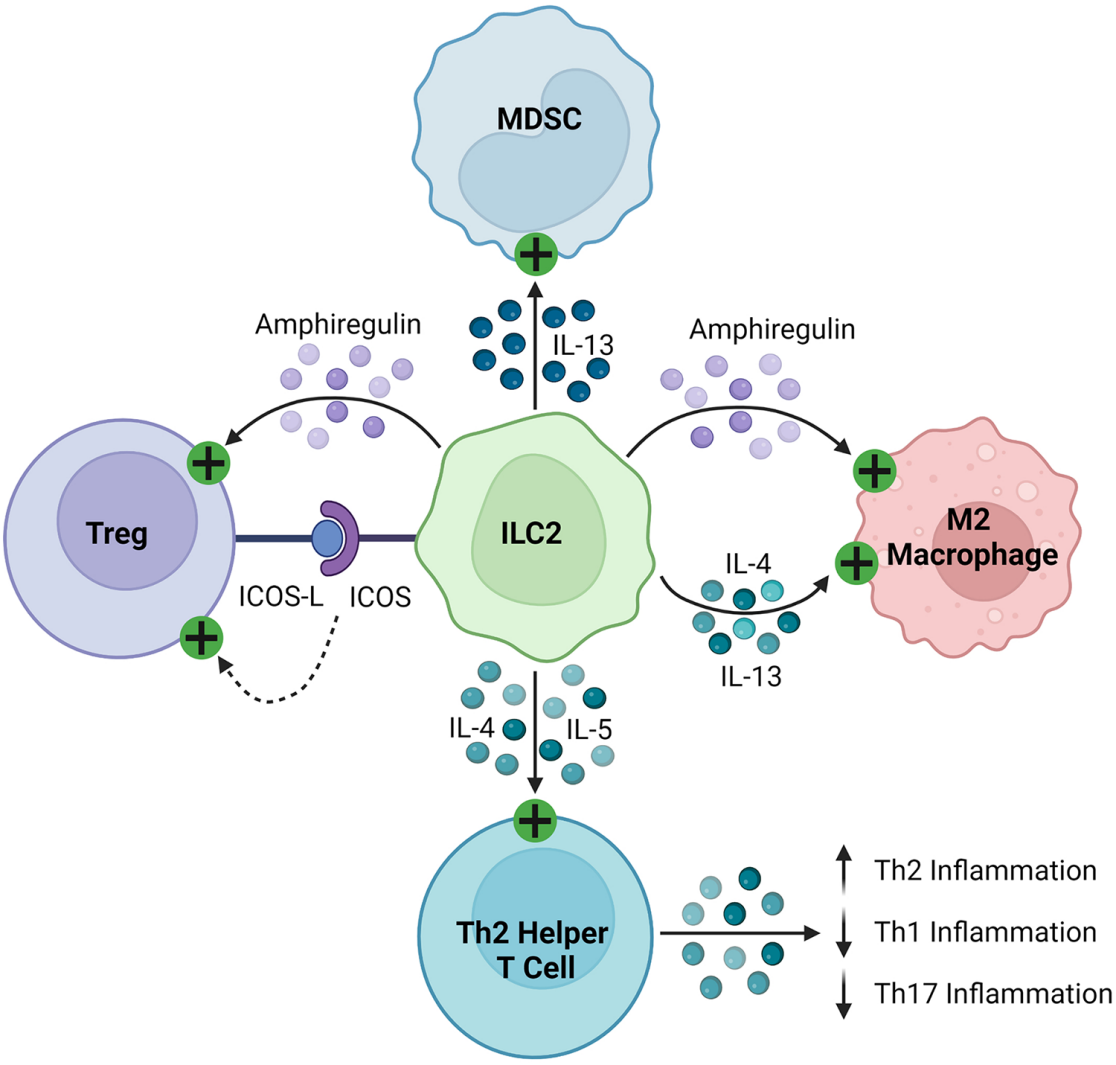
Immunosuppressive mechanisms of ILC2s. ILC2s express ICOS and activate Tregs by binding their cognate ICOS-L. They secrete amphiregulin, which activates Tregs and M2 macrophages. ILC2s produce IL-4, IL-5, and IL-13, which then stimulate MDSCs, M2 macrophages, and Th2 helper T cells, promoting a Th2 polarized inflammatory response.

ILC2s regulate the immune response through the production of soluble proteins and direct cell-cell interactions, as seen in Figure 4. They interact with T cells, firstly through the production of various effector cytokines, including IL-4, IL-5, and IL-13, to promote type 2 inflammation (143–146, 148, 155). As early and potent sources of these cytokines, ILC2s recruit and activate Th2 helper T cells to sites of inflammation, stimulating a positive feedback loop and suppressing more damaging Th1 and Th17 inflammation (165, 166). Like other innate immune cells described in this review, ILC2s can activate Tregs through the production of amphiregulin and direct cell contact, including signaling through inducible co-stimulator (ICOS)/IOCS ligand (ICOSL) and GITR/GITRL binding (160, 163, 167). In various cancers, ILC2s have been found to inhibit the anti-tumor immune response by promoting the infiltration and activation of MDSCs via IL-13 signaling (159, 168, 169). They have also been shown to stimulate and maintain M2 macrophages in inflamed tissues, which are less inflammatory than their M1 counterparts, through IL-5 and IL-13 signaling (157, 161).

Finally, ILC2s play a protective role in tissue repair, proliferating in response to alarmins, such as IL-33 and IL-25, released by the damaged epithelium after tissue injury or ischemia (154, 164, 170, 171). ILC2s subsequently upregulate their signature cytokines and amphiregulin, the latter of which controls the expansion and differentiation of various cell types through epidermal growth factor receptor signaling, to promote repair of the damaged epithelium (146, 172). To further support their potential role in solid organ transplantation, recent studies have illustrated that ILC2s attenuate IRI through M2 macrophage activation by IL-4, IL-13, or amphiregulin (164, 173).

There is limited correlational data regarding ILC2s in transplant patients, with one single-center cohort study demonstrating an inverse relationship between the number of ILC2s in lung allografts following reperfusion and the risk of primary graft dysfunction (174). While they are the least extensively studied cell type in this review and will require further investigation to elucidate their role in solid organ transplantation, a few preclinical studies have shown that ILC2s can be expanded *in vitro* and adoptively transferred to shape the alloimmune response. These studies will be covered in the next section.

## 3. Engineering tolerance and the development of innate immune cellular therapeutics

Based on the immunosuppressive properties of these innate immune effector cells *in vivo*, they have been proposed as therapeutic targets either to reduce reliance on immunosuppressive drugs or modulate peripheral tolerance through interactions with other immunoregulatory cells. As they are generally not present in sufficient numbers during the alloimmune response, however, they require stimulation or modulation *ex vivo* to exert robust immunosuppressive effects. In this section, we summarize preclinical studies applying *in vitro*-generated innate immune cells to models of transplantation, as well as clinical trials that have been conducted in transplant patients to date.

### 3.1. Myeloid-derived suppressor cells

MDSCs develop from immature myeloid cells in response to signals of chronic inflammation, including granulocyte colony-stimulating factor (G-CSF), granulocyte-macrophage colony-stimulating factor (GM-CSF), TNF-α, IFN-γ, TGF-β, lipopolysaccharide (LPS), CXCL-1/2, IL-2, and IL-6 (8, 9, 18, 175–179). Previous work has demonstrated that MDSCs can be induced *in vitro* from human bone marrow precursors cells in the presence of IL-6, GM-CSF, and G-CSF (179). Their subsequent immunosuppressive capacity, however, varies based on the cytokines used, with the greatest effect observed with combination GM-CSF and IL-6 treatment (179).

MDSCs have been shown to delay allograft rejection in multiple animal models, as summarized in Table 1. As mentioned above, activated MDSCs have been generated in culture using cytokines such as IL-6, G-CSF, and GM-CSF or induced *in vivo* with G-CSF or IL-33, both of which promoted skin allograft tolerance (36, 40, 175, 179–184). MDSCs generated *in vitro* with GM-CSF and IL-6 successfully delayed islet allograft rejection up to 200 days through CD8^+^ T cell suppression and NO production, the latter of which promoted antigen-specific Treg expansion and migration to lymphoid organs near the allograft (26, 37, 179). Lastly, MDSCs prolonged the survival of vascularized allografts in rodents following adoptive transfer or induction with agents such as IL-33 and anti-CD40L monoclonal antibody for co-stimulation blockade (14, 185, 186).

**Table 1:** Studies of MDSCs in animal models.

Organ	Species	Source	Induction Agent(s)	Adjuncts	Mean or Median Allograft Survival^a^	Mechanism	Ref
Skin	Mouse	Recipient (36, 40, 175, 180–183) Donor (36)	G-CSF (182) GM-CSF (175, 181) M-CSF (36, 180) IL-6 (175, 181) IL-33 (183) IFN-λ (36) LPS (40, 175) TNF-α (180)	N/A	45 vs. 23.5 (181) 40 vs. 16 (182)	HO-1 (40) iNOS (36, 180) IL-10 (40) Treg expansion (181) Effector T cell apoptosis (181) T cell anergy (182)	(36, 40, 175, 180–183)
Islet	Mouse	Recipient (26, 37, 179)	G-CSF (179) GM-CSF (37, 179) IL-4 (37) IL-6 (179) Hepatic stellate cells (26, 37)	N/A	N/A	C-EBPβ (179) iNOS (37) Activation of Tregs (26)	(26, 37, 179)
Heart	Mouse	Recipient (14, 184, 185)	IL-33 (184) Ethyl-carbodiimide treated donor splenocytes (185)	anti-CD40L mAb (14)	29 vs. 9 (184)	IDO (185) iNOS (14, 185)	(14, 184, 185)

^a^ Experimental versus control, days.

There are no clinical trials of MDSCs in transplant patients to date. While it remains unclear why they have not been pursued more aggressively as cellular therapeutics in these patients, the inherent challenges of generating immature immune cells, such the risk of differentiation once removed from artificial culture conditions and difficulties tracking their reconstitution *in vivo*, are likely contributing factors. Additional studies examining methods to expand these cells *in vivo* may yield more durable results.

### 3.2. Human monocyte-derived suppressor cells

Several studies have described the anti-inflammatory effects of human monocyte-derived suppressor cells (HuMoSCs) in murine models of graft-versus-host disease (GVHD) (187–189). HuMoSCs share many features with human M-MDSCs, as they are generated *in vitro* from PBMCs in the presence of GM-CSF and IL-6 and are defined as CD11b^+^ CD14^+^ CD33^+^ cells, but notably differ based on the former's high expression of HLA-DR (189). Using a xenogenic model of GVHD in which human PBMCs were injected into immunocompromised NSG mice, Janikashvili et al. demonstrated that concurrent infusion of autologous HuMoSCs could prevent GVHD and improve overall survival (189). Interestingly, these HuMoSCs inhibited both autologous and allogenic effector T cells *in vitro* and promoted expansion of CD8^+^ Tregs *in vivo*, the former of which is particularly relevant for targeting donor and recipient T cell contributions to the alloimmune response (189). In a follow up study, HuMoSCs retained their immunosuppressive capacity in a proinflammatory cytokine milieu and demonstrated an enhanced survival benefit in GVHD with the concurrent administration of cyclophosphamide (187). Given difficulties generating large numbers of HuMoSCs *in vitro* due to limited yield from PBMCs with existing protocols, administration of their culture supernatant as a therapeutic was trialed and found to alleviate xenogenic GVHD in mice, thought to be mediated by various immunosuppressive proteins including IL-1RA, GPNMB, and galectin-3 (188). Together, these studies support the feasibility of generating immunosuppressive myeloid cells from human PBMCs and the efficacy of the cells themselves or their products, which is encouraging for such application of HuMoSCs and MDSCs (187–189).

### 3.3. Regulatory dendritic cells

While the optimal method for generating stable regDCs remains a subject of debate, they are commonly induced *in vitro* from bone marrow cells in rodents and PBMCs in human subjects by culturing with GM-CSF with or without IL-4, followed by an anti-inflammatory or immunosuppressive agent to arrest further maturation (49, 69, 190–197). Such agents include anti-inflammatory cytokines (such as TNF-α, TGF-β, or IL-10), immunosuppressive drugs (such as mycophenolate, rapamycin, or corticosteroids), vitamins (such as vitamin D3 or retinoids), or tissue factors (such as vasoactive intestinal peptide or hepatocyte growth factor) (49, 69, 190–197).

The immunoregulatory properties of regDCs were first investigated in other inflammatory conditions, particularly autoimmune disorders following the observation that deletion of DCs induces spontaneous autoimmunity in mice (48, 190, 198). Preclinical studies demonstrated a protective role for *in vitro*-generated regDCs in such diseases, including rheumatoid arthritis and Crohn's disease, and more recently their short-term safety and efficacy were supported in clinical trials of patients with these disorders, type 1 diabetes mellitus, and multiple sclerosis (199–206). RegDCs also attenuated GVHD following bone marrow transplantation in mice (190). Based on these findings, multiple studies have investigated donor-derived regDCs in preclinical transplant models, as summarized in Table 2 (193, 207–216).

**Table 2:** Studies of regDCs in animal models.

Organ	Species	Source	Induction Agent(s)	Adjuncts	Mean or Median Allograft Survival^a^	Mechanism	Ref
Skin	Mouse	Recipient	GM-CSF	N/A	31 vs. 23.5	T cell anergy	(181)
Islet	Mouse	Recipient (193, 217)	1, 25(OH)_2_D_3_ (193) GM-CSF (217)	MMF (193) anti-CD3 Ab (217)	> 70 vs. 23 (193) 77.4 vs. 19.6 (217)	Activation of Tregs (193) Treg expansion (217)	(13, 193)
	Rat	Recipient	GM-CSF IL-4	ALS	> 200 vs. 10.3	Acquired thymic tolerance via indirect pathway	(218)
Heart	Mouse	Donor (207, 209–212, 219) Recipient (220)	GM-CSF (207, 210–212, 219, 220) IL-4 (207, 211, 212, 220) IL-10 (209) TGF-β (209–212) Rapa (220)	CTLA-4 Ig (209, 210, 219) anti-CD40L mAb (211, 212) anti-ICAM mAb (219) FK506 (220)	> 100 vs. 8 (207) 29 vs. 11.1 (209) 71 vs. 10 (210) 77 vs. 12 (211) > 100 vs. 20 (219) 46.8 vs. 9.1 (220)	IL-10 (209) T cell anergy (207, 211, 219, 220) Inhibition of T cell proliferation (209) Apoptosis of effector T cells (210, 212) Treg expansion (209)	(207, 209–212, 219, 220)
	Rat	Donor (208, 221, 222) Recipient (62, 223–225)	GM-CSF (62, 208, 221–225) IL-4 (221–225) Dex (222)	ALS (221, 223) CSA (222) CTLA-4 Ig (222) Rapa (225) LF 15-0195 (225)	> 100 vs. 10.2 (223) 16.5 vs. 6 (224) 100 vs. 6 (225)	HO-1 (62) T cell anergy (221) Activation of Tregs (222) Acquired thymic tolerance via indirect pathway (223) iNOS (224)	(62, 82, 208, 221–224)
Kidney	Rat	Donor	GM-CSF IL-4 Dex	CTLA-4 Ig CSA	N/A	Treg expansion	(222)
	Rhesus macaque	Donor (215, 216) Recipient (226)	1, 25(OH)_2_D_3_ (215, 216, 226) IL-4 (226) IL-10 (215, 216, 226) GM-CSF (226)	CTLA-4 Ig (215, 216, 226) Rapa (215, 216, 226)	113.5 vs. 39.5 (216) 56 vs. 39.5 (226)	Memory T cell exhaustion (215, 216) Effector T cell exhaustion (226) Suppression of Th17 inflammatory response (226)	(215, 216, 226)
CTA	Rat	Donor (213) Recipient (214)	GM-CSF (213) Rapa (213) Cell-free donor spleen lysate (213) IL-10 (214)	CSA (213) ALS (213) FK506 (214)	98.5 vs. 10 (213) 46.7 vs. 5 (214)	T cell anergy (213) IL-4 (214) IL-10 (213, 214)	(213, 214)

ALS, anti-lymphocyte serum; CSA, cyclosporine; Dex, dexamethasone; MMF, mycophenolate mofetil; FK506, tacrolimus; Rapa, rapamycin; 1, 25(OH)_2_ D_3_, vitamin D_3_.

^a^ Experimental versus control, days.

Preoperative or postoperative administration of a single dose of donor-derived regDCs prolonged heart allograft survival beyond 100 days in mice and rats, with these regDCs inhibiting T cell responses both *in vitro* and *in vivo* (207, 208). Interestingly, Lan et al. observed that the effects of regDCs could be potentiated with concurrent administration of CTLA-4 immunoglobulin (Ig), further delaying allograft rejection, while Bohnam et al. described similar results after genetically modifying regDCs to express CTLA-4 Ig (209, 210). Additional studies demonstrated a synergistic effect between regDCs and low dose immunosuppression in prolonging cardiac and renal allograft survival (211, 212, 217–219). Furthermore, donor-derived regDCs have successfully induced tolerance of rodent renal and composite tissue (CTA) allografts with standard-of-care immunosuppressants (193, 213, 214, 218). Donor-derived regDCs were then investigated in non-human primates (NHPs), with a single infusion of cells extending allograft survival nearly threefold to a median 113.5 days (from 39.5 days in controls) following MHC-mismatched kidney transplantation in rhesus macaques with minimal immunosuppression (216). Of note, these regDCs were found to have no adverse effects and induced substantial donor-specific memory T cell exhaustion (215, 216).

Given that donor-derived regDCs would be largely limited to use in living donor transplants and could cause sensitization, additional efforts have been dedicated to developing recipient-derived (or autologous) regDCs as cellular therapeutics (220–227). Autologous regDCs pulsed with donor allopeptides were found to delay islet, cardiac, and CTA rejection in rodents, likely through the indirect allorecognition pathway (220–222). As seen with donor-derived regDCs, the effects of autologous regDCs could be potentiated by an adjunct, as Baas et al. described superior islet allograft survival with concurrent anti-CD3 antibody administration (221, 222). Autologous regDCs promote tolerance in an antigen-specific manner by inducing anergy of alloreactive T cells and proliferation of Tregs *in vivo* in various transplant models, while syngeneic regDCs without alloantigen exposure delay rejection in a non-specific manner through NO production (224–226). Furthermore, allopeptide-pulsed autologous regDCs led to modest delays in MHC-mismatched renal allograft rejection in NHPs to 56 days (from 39.5 days in controls with no infusion and 29 days with naïve regDC infusion) in an HO-1-dependent manner (62, 227).

RegDCs are undergoing investigation in a few transplant clinical trials (228). The ONE Study (NCT02252055) tested multiple regulatory cell products in living donor renal transplant recipients in seven separate study arms, with one arm investigating one-time infusion of non-pulsed autologous regDCs one day prior to transplantation followed by standard-of-care immunosuppression (228). In this phase 1/2 trial, aggregate analysis demonstrated these regulatory immune cell products, including regDCs, were safe, feasible, associated with fewer viral infections, and led to successful weaning of immunosuppression in many participants at one year post-transplant (228). Phase 1/2 clinical trials of donor-derived regDCs in living donor kidney and liver transplant patients are ongoing at the University of Pittsburgh (NCT03726307, NCT03164265). Similar to the ONE Study, investigators will attempt to wean immunosuppression in these patients starting at six months post-transplant in the absence of rejection (228). Additional studies and clinical trials will clearly be needed to compare the efficacy of donor- and recipient-derived regDCs, the optimal dosing and timing of administration, and their long-term effects in transplant patients.

### 3.4. Regulatory macrophages

Mregs may be generated from bone marrow precursors or PBMCs *in vitro* following the activation of two signals. The first signal initiates the polarization of monocytes to monocyte macrophages, which then activate Mregs, and can be triggered by agents such as growth factors [including GM-CSF and macrophage colony-stimulating factor (M-CSF)], apoptotic cells, and glucocorticoids (78, 87, 229–232). With the second signal, Mregs are directly activated by TLR ligands and cytokines, which leads to downregulation of inflammatory factors and upregulation of inhibitory factors (95, 233–235). Mregs have been successfully generated from PBMCs in culture using M-CSF, human serum, and a brief 24-h pulse of IFN-γ, and while this approach has been utilized in clinical trials with renal transplant patients, their optimal induction method remains unclear (86, 92).

Mregs have been shown to delay allograft rejection in multiple preclinical models, as demonstrated in Table 3 (55, 83, 181, 236–243). Firstly, CSF1 treatment attenuates GVHD following bone marrow transplantation in mice through the expansion of recipient Mregs that inhibit allogenic donor T cell responses (236). Subsequent adoptive transfer experiments demonstrated preoperative donor-derived Mreg infusion delays rejection of skin, CTA, and heart allografts, the latter in an iNOS-dependent fashion enhanced by concurrent rapamycin administration (83, 181, 240, 243). In contrast to regDCs discussed above, only donor-derived Mregs are effective in delaying rejection, with no benefit observed with autologous Mregs (55, 83). In heart transplant recipients treated with anti-CD40L mAb costimulatory blockade, allograft tolerance was associated with enhanced migration of Ly6C^hi^ monocytes to the graft, which then differentiated to Ly6C^lo^ Mregs that secreted IL-10 in response to DC-SIGN or TLR4 signaling (237–239). In a porcine lung transplant model, donor-derived Mreg infusion did not prolong allograft survival, perhaps due to an insufficient dosage of cells (241). Finally, *in vitro* studies found that human Mregs can suppress the xenogenic immune response to porcine cells in an IDO-dependent manner, suggesting a potential role for these cells in xenotransplantation (242).

**Table 3:** Studies of Mregs in animal models.

Organ	Species	Source	Induction Agent(s)	Adjuncts	Mean or Median Allograft Survival^a^	Mechanism	Ref
Heart	Mouse	Donor	M-CSF, IFN-γ	Rapa, MMF	66.3 vs. 8.7	iNOS	(83)
CTA	Rat	Donor	M-CSF, IFN-γ	N/A	7.7 vs. 5.7	N/A	(240)
Lung	Pig	Donor	M-CSF, IFN-γ	MP, FK506, preoperative XRT	307 vs. 92	N/A	(241)

FK506, tacrolimus; MMF, mycophenolate mofetil; MP, methylprednisolone; Rapa, rapamycin; XRT, preoperative radiation therapy.

^a^ Experimental versus control, days.

Mregs are the most extensively studied innate immune cells in transplant clinical trials (86, 228, 244–247). They were first investigated in the phase 1 Transplant Acceptance-Inducing Cell trial I (TAIC-I) in which deceased donor kidney transplant patients received one infusion of donor-derived Mregs on postoperative day five (244). No adverse effects were reported and two patients successfully weaned from standard immunosuppression, but the therapeutic benefit of these cells could not be clearly discerned (244, 245). TAIC-I was followed by several small phase 1 trials of preoperative Mreg administration in living donor kidney transplantation at various time points, with six out of eight patients transitioning to tacrolimus monotherapy without eliciting rejection (86, 246, 247). Of the two remaining patients, one was undergoing living-related kidney transplant against which he was already sensitized but was subsequently transitioned to low dose tacrolimus and prednisolone with no rejection episodes (247). Interestingly, at eight weeks postoperatively, he was found to be hyporesponsive to his donor on mixed lymphocyte reaction and had resolved his donor-specific antibodies, which remained absent through the 53 week follow up period (247). As previously mentioned, the ONE Study (NCT02085629) investigated various regulatory immune cells in living donor renal transplant patients, with one arm dedicated to donor-derived Mregs (228). Administration of these cells, including Mregs, led to no adverse events and was associated with a reduction in post-transplant viral infections and successful weaning of immunosuppression (228). Overall, significant preclinical and early clinical trial data suggest donor-derived Mregs may be harnessed to prevent rejection in solid organ transplant patients.

### 3.5. Natural killer cells

In the setting of transplantation, NK cells have been found to participate in both rejection and tolerance, likely due to the distinct functions of their various subsets or differentiation states (248). Despite these mixed findings, several preclinical studies support a role for NK cells in inducing allograft tolerance (110, 111, 113, 121–123, 249, 250). Firstly, NK cells were shown to attenuate the severity of GVHD in mice by suppressing activated alloreactive T cells, mediated by perforin and FasL signaling (249). As mentioned in the prior section, several studies have found that tolerance induction by costimulatory blockade is dependent on NK cells (121, 122). Various functions of NK cells have been implicated in this phenomenon, including perforin secretion in an islet allograft model and NKG2D receptor signaling in a heart allograft model (121, 122). Additionally, NK cells were shown to delay skin graft rejection through cytotoxic killing of donor-derived APCs, which would otherwise activate host alloimmune T cells (110, 111). NK cells also delayed rejection of murine skin grafts by suppressing alloimmune T cell responses directly, either through competition for shared growth factors or cytotoxic killing (113, 250). Finally, upregulation of the NKG2A receptor in NK cells following islet transplantation stimulated secretion of IL-22, which attenuated inflammation and prolonged allograft survival (123). These studies are summarized in Table 4.

**Table 4:** Studies of NK cells in animal models.

Organ	Species	Source	Induction Agent(s)	Adjuncts	Mean or Median Allograft Survival^a^	Mechanism	Ref
Skin	Mouse	Recipient	Depletion with anti-NK1.1 mAb (110, 111, 250) Perforin knockout (110, 113)	CTLA-4 Ig (110, 113) Anti-CD40L mAb (111) Anti-OX40L mAb (111) Anti-gp39 mAb (113)	> 80 vs. 16 (113) 15 vs. 12 (250)	Cytotoxic killing of donor APCs (110, 111) Cytotoxic killing of effector T cells (113) Inhibition of cytotoxic CD8^+^ T cells (250)	(110, 111, 113, 250)
Islet	Mouse	Recipient (121, 123)	Depletion with anti-NK1.1 mAb (121) Perforin knockout (121)	Anti-CD40L mAb (121)	N/A	Perforin (121) Upregulation of NKG2D (123) IL-22 (123)	(121, 123)
Heart	Mouse	Recipient	anti-NKG2D Ab	CTLA-4 Ig	47.5 vs. 22.5	NKG2D signaling	(122)

^a^ Experimental versus control, days.

Finally, with recent advances in genetic modification of immune cells, chimeric antigen receptor (CAR)-NK cells have been developed as novel therapeutics for advanced malignancies resistant to standard treatment (251, 252). Unlike CAR-T cells, CAR-NK cells can be generated from individuals other that the recipient without the risk of GVHD and have not been associated with common side effects of the former, such as neurotoxicity or cytokine release syndrome (253–255). These features are promising for their application as “off-the-shelf” cellular products and with greater understanding of the inherent immunosuppressive features of these cells, we imagine such engineered NK cells may be explored as therapeutics to prevent or treat rejection in the coming years. In summary, preclinical studies suggest a subpopulation of NK cells can modulate the alloimmune response to promote allograft tolerance, and recent advances in immunotherapy support the feasibility and efficacy of engineered NK cells as therapeutics.

### 3.6. Innate lymphoid cells

Preclinical studies investigating ILC2s in transplantation are limited to date, as summarized in Table 5. Bruce et al. found that adoptive transfer of activated ILC2s attenuated the severity and mortality of GVHD in mice, as they migrated to the gastrointestinal tract to improve barrier function and recruited MDSCs to suppress inflammation (256). Perhaps most exciting, Huang et al. demonstrated that systemic IL-33 treatment or infusion of IL-33-primed ILC2s could significantly delay rejection of islet allografts with major antigen mismatch via IL-10 secretion (257). The efficacy of IL-33 treatment was dampened following depletion of Tregs, which themselves are stimulated by IL-33 and have been shown to delay allograft rejection in other transplant models (185, 257–260). Furthermore, IL-33 is also known to activate MDSCs to delay heart allograft rejection in mice, which was not investigated by the authors (185, 257). Donor-derived ILC2s activated by IRI were recently shown to enhance eosinophil recruitment to the allograft following lung transplantation, leading to reduced T cell infiltration and attenuating rejection at seven days post-transplant (261). Of note, this axis was dependent on donor ILC2s, rather than infiltrating recipient ILC2s, suggesting additional work will be required to differentiate the roles of donor and recipient ILC2s in other transplant models (261). These studies are reviewed in Table 5.

**Table 5:** Studies of ILC2s in animal models.

Organ	Species	Cell Source	Induction Agent(s)	Adjuncts	Mean or Median Allograft Survival^a^	Mechanism	Ref
Islet	Mouse	Recipient	IL-33, IL-2 complex	N/A	N/A	IL-10	(256)
Lung	Mouse	Donor	IL-33	CSA/MP	N/A	IL-5 mediated eosinophil recruitment	(257)
				CTLA-4 Ig			
				Anti-CD40L mAb			

CSA, cyclosporine; MP, methylprednisolone.

^a^ Experimental versus control, days.

Overall, our current understanding of ILC2s suggests they may be capable of inhibiting the alloimmune response and preventing allograft rejection through multiple pathways, including stimulating tissue repair, secreting anti-inflammatory cytokines, and activating other immunosuppressive cells (146, 154, 156–164). Additional investigation, however, is clearly needed to characterize the functions of ILC2s in different tissues, their roles when derived from donor versus recipient, and their long-term effects in human transplant patients.

## 4. Discussion

Preclinical and clinical studies performed to date investigating the therapeutic potential of innate immune cells to prevent or treat allograft rejection are encouraging. Infusion of MDSCs, HuMoSCs, regDCs, Mregs, NK cells, and ILC2s may eventually reduce or replace the need for non-specific, chronic immunosuppression, with early clinical trials of Mregs and regDCs demonstrating successful weaning in some patients (86, 228, 244, 246). Many questions, however, remain regarding their use, including the most appropriate dosing range, timing, and frequency of administration. Weekly adoptive transfers of MDSCs, for example, led to superior allograft survival than a single administration following skin transplantation in mice (175). Mreg infusion has been trialed both preoperatively and postoperatively in small clinical studies of kidney transplant patients, with no clear conclusion yet as to the best strategy (86, 244, 246, 247).

Furthermore, additional studies are needed to elucidate the optimal source of these cells. While only donor-derived Mregs promote tolerance, both donor- and recipient-derived regDCs have been utilized effectively in animal models (55, 83, 207, 208, 220, 224, 225). Given the breadth of signals that drive the proliferation and maturation of these cells, it will be necessary to determine the optimal conditions for their induction *in vitro*, as has been investigated for MDSCs and Mregs to some extent (85, 86, 92, 179). Given that these cells are known to interact with each other and other immunosuppressive cells, whether infusion of multiple cell types potentiates their effects *in vivo* warrants further investigation as well (14, 26, 64–67, 159, 168, 169). Similarly, some studies have demonstrated greater efficacy of these cells when administered with an adjunct, such as CTLA-4 Ig with regDCs and rapamycin with Mregs, which should be explored further (83, 209, 210).

Finally, recent studies suggest that innate immune cells may be targeted *in vivo* to promote transplant tolerance by stimulating or inhibiting various signaling pathways, including microRNA (miRNA) and purinergic signaling (262–265). Usuelli et al. found that suppression of miRNA-21 prevented allograft rejection and chronic allograft vasculopathy in a murine model of cardiac transplantation by promoting M2 polarization in infiltrating macrophages (262). Conversely, other groups have shown that miRNA-22 is critical for the development of functional MDSCs and M2 macrophages following CSF1R signaling (263, 264). Additional work will be needed to elucidate the roles of these various soluble factors and signaling pathways in the alloimmune response, and to determine which pathways could be selectively targeted with novel therapeutics in these various regulatory innate immune cells to promote tolerance.

Large scale clinical trials will be necessary to answer these questions prior to the widespread implementation of innate immune cells as therapeutics in transplantation. Long-term follow up will be critical to characterize any adverse events or unintended consequences of these cells over time, such as possible fibrosis associated with prolonged ILC2 activation (266, 267). Overall, innate immune cells represent a promising new therapeutic strategy to induce tolerance following solid organ transplantation and we look forward to their translation to clinical practice.

## Author contributions

LCO and AGC contributed to the writing of this manuscript. All authors contributed to the article and approved the submitted version.
